# Pretreatment Blood Markers in the Prediction of Occult Neck Metastasis: A 10-Year Retrospective Study

**DOI:** 10.7759/cureus.16641

**Published:** 2021-07-26

**Authors:** Eduardo Ventura, João Barros, Inês Salgado, Ana Millán, Miguel Vilares, Carlos Zagalo, Pedro Gomes

**Affiliations:** 1 Oral and Maxillofacial Surgery Unit, Centro Hospitalar e Universitário do Porto, Porto, PRT; 2 Oral and Maxillofacial Surgery Department, Centro Hospitalar e Universitário de Coimbra, Coimbra, PRT; 3 Department of Head and Neck Surgery, Instituto Português de Oncologia de Lisboa Francisco Gentil, Lisboa, PRT; 4 Centro de Investigação Interdisciplinar Egas Moniz (CiiEM), Egas Moniz - Cooperativa de Ensino Superior, CRL, Monte da Caparica, PRT

**Keywords:** occult neck metastasis, neutrophil-lymphocyte ratio, depth of invasion, squamous cell carcinoma, oral cancer

## Abstract

Introduction

The purpose of this study was to clarify the role of inflammatory blood markers in the management of early-stage (T1-T2) oral squamous cell carcinoma (OSCC) of the tongue in patients with a clinically negative neck.

Materials and methods

We undertook a retrospective chart review of 102 patients with early-stage OSCC of the tongue, subjected to tumor resection and elective neck dissection. Based on postsurgical histopathological examination results, we divided our cohort into pN+ and pN0 groups. Afterwards, we analyzed the role of pretreatment inflammatory blood markers in predicting occult neck metastasis. We also evaluated neutrophil-lymphocyte ratio (NLR) association with the depth of invasion (DOI) of the primary tumor.

Results

We found a significant association of NLR (p=0.001) and monocyte-lymphocyte ratio (p=0.011) with neck status on univariate analysis. Multivariate analysis showed that only NLR (p=0.02) was an independent risk factor for occult metastasis among inflammatory blood markers. Receiver Operating Characteristic curve analysis and Younden’s Index determined the NLR value of 2.96 as the most adequate cut-off value for neck status prediction. NLR values of pretreatment workup also had a significant association with the DOI of the primary tumor (p=0.018).

Conclusion

Our study supports the role of pretreatment NLR in predicting occult neck metastasis in early-stage OSCC of the tongue. It also sheds some light over the potential of NLR as a predictor of the primary tumor’s DOI.

## Introduction

Oral squamous cell carcinoma (OSCC) is one of the most common cancers worldwide [1,2], and the tongue remains its most common subsite [2,3]. Since the most important factor in the prognosis of OSCC is the status of the regional nodes, and since the presence of nodal metastasis decreases patients’ survival by nearly 50%, an adequate evaluation of neck disease by the surgeon is critically important [4,5].

The management of patients without clinical disease of the neck (cN0) has long been debated [2,5]. With occult nodal metastasis in early-stage OSCC tumors (T1-T2) ranging from 20% to 40% [2], choosing not to perform an elective neck dissection (END) may lead to suboptimal oncological outcomes [6]. Therefore, there has been a growing trend in the literature towards upfront END as opposed to a “watch and wait” policy in cN0 patients [2,7]. On the other hand, there has also been a clear tendency to regard tumor Depth of Invasion (DOI) as the most predictive parameter for nodal metastasis and to consider sentinel node biopsy in decision making, mainly in clinically “thin” OSCC (DOI <4 mm) [2,7]. Unfortunately, preoperative DOI estimation cannot always be guaranteed with accuracy [2], and sentinel node biopsy is a technically demanding procedure, whose success rates depend on local technical expertise and experience [7]. Consequently, any additional tools that could be developed to clarify OSCC cN0 patients’ management will most likely lead to better clinical outcomes. 

Systemic inflammation is a major contributing factor in cancer development and progression across several types of tumors [8]. Numerous studies have shown that changes occur across blood cell lines, and their ratios, during carcinogenesis [9]. Inflammatory blood markers like neutrophil-lymphocyte ratio (NLR), monocyte-lymphocyte ratio (MLR), platelet-lymphocyte ratio (PLR) red cell width distribution (RDW), mean platelet volume (MPV) and fibrinogen values have been described as prognostic factors for several malignancies [10-17]. Some authors have even demonstrated that NLR could play a role in predicting occult nodal metastasis in early-stage tongue tumors [3,6].

We aim to confirm the role of NLR in the prediction of occult neck metastasis in early-stage OSCC of the tongue patients’ with cN0 and to assess the usefulness of other blood markers on that endeavour.

## Materials and methods

We have carried out a retrospective review of the clinical records of all the patients with early-stage OSCC of the tongue, subjected to resection of the primary tumor and END, between the 4th of January 2010 to the 17th of January 2020, at Instituto Português de Oncologia de Lisboa, a tertiary referral center and one of the most important cancer centers in our country. The study was undertaken in compliance with the Helsinki Declaration. Data collection took place from January 2020 to March 2020.

We applied the following inclusion criteria: confirmation of OSCC of the tongue through a preoperative incisional biopsy; postoperative confirmation of OSCC of the tongue, with a maximum diameter of 40 mm; cN0 at the preoperative stage, without any clinical or imagiological evidence of regional or distant metastasis.

The defined exclusion criteria were: recurrence of the primary tumor; clinical history of chemotherapy or radiotherapy; clinical history of other tumors; clinical conditions or medication history that potentially could lead to changes in inflammatory markers (infection, chronic inflammatory or auto-immune diseases, hematologic disorders, treatment with steroid or non-steroid anti-inflammatory drugs, anticoagulants or antiplatelet agents).

The data available on the factors predisposing to malignancy (smoking, alcohol consumption and infection by human papillomavirus) was incomplete. Therefore we did not take these into account in our study.

As a standard, all patients’ pretreatment workup included complete physical examination, routine blood counts, liver functions tests, maxillofacial computed tomography (CT), CT or US (ultrasonography) of the neck, and chest CT. All CTs were performed with contrast enhancement. 

**Figure 1:**
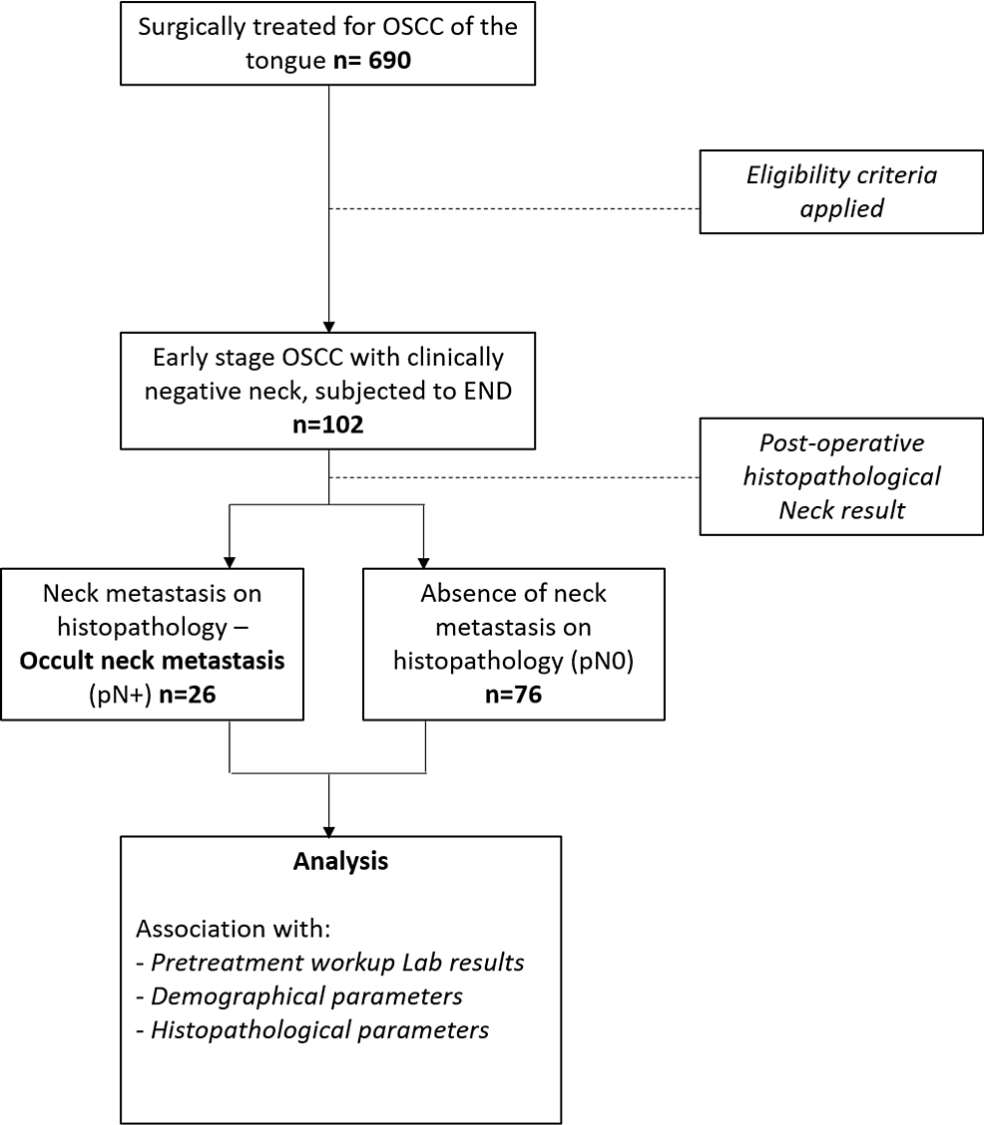
Study flowchart.

According to postoperative histopathology, we divided our subjects into two groups, based on the presence (pN+ group) or absence (pN0 group) of neck metastasis. Afterwards, we studied the association of the demographic parameters, pretreatment blood markers and postoperative histopathological results of our sample, with neck status classification (pN+ or pN0) (Figure 1).

We retrieved the inflammatory pretreatment blood markers from the routine blood counts of pretreatment workup. NLR was obtained as the “absolute neutrophil count” divided by the “absolute lymphocyte count”. MLR was determined as the “absolute monocyte count” divided by the “absolute lymphocyte count”. PLR was calculated as the “absolute platelet count” divided by the “absolute lymphocyte count”. We also analyzed the association of RDW, MPV, and fibrinogen results with neck status classification.

We used SPSS 27.0.1 and Jamovi 1.1.9.0 software for statistical analysis. Student’s t and Mann-Whitney U tests were applied for univariate analysis of the association of continuous variables and neck status classification. Shapiro-Wilk was used as a normality test and Levene test was used to assess the equality of variances. The Chi-square test was used for the univariate analysis of categorical variables with neck status classification. We defined a p-value <0.05 for the determination of statistical significance in all statistical tests.

Logistic regression with the stepwise regression method was applied for multivariate analysis of risk factors.

Receiving operating characteristic (ROC) curve analysis, with area under the curve and Younden index calculation, was employed to evaluate the discriminatory performance of NLR in assessing neck status classification.

As a secondary aim of study, we also conducted a univariate analysis of the association of NLR with the primary tumor DOI.

## Results

Of the 690 patients that were surgically treated for OSCC of the tongue at Instituto Português de Oncologia de Lisboa, during the aforementioned period, 588 patients were excluded after applying eligibility criteria.

The 102 patients who fulfilled the eligibility criteria were treated with surgical resection of the primary tumor and END. Eleven of these patients were subjected to bilateral END, because of primary tumor’s proximity to the midline. Regarding neck status, according to postoperative histopathological examination, 26/102 (24.5%) were positive for neck metastasis (pN+) while 76/102 (75.5%) were negative for neck metastasis (pN0) (Table 1).

**Table 1:** Demographical, histopathological and laboratory parameters of our cohort of patients. y: years; SD: standard deviation; NLR: neutrophil-lymphocyte ratio; MLR: monocyte-lymphocyte ratio; PLR: platelet-lymphocyte ratio.

Cohort parameters	
Gender	
Male - n (%)	63 (61.8)
Female n (%)	39 (38.2)
Age at time of surgery, y	
Mean (range)	60.1 (24-89)
Cervical metastasis	
Absent pN0 - n (%)	76 (74.5)
Present pN+ - n (%)	26 (25.5.)
Tumour dimension	
p<2 cm - n (%)	38 (37.3)
p 2-4 cm - n (%)	64 (62.7)
Perineural/perivascular invasion	
Yes - n (%)	25 (24.5)
No - n (%)	77 (75.5)
Differentiation grade	
G1 - n (%)	20 (19.6)
G2 - n (%)	71 (69.6)
G3 - n (%)	11 (10.8)
NLR	
Mean (SD)	2.2 (1.3)
MLR	
Mean (SD)	0.2 (0.1)
PLR	
Mean (SD)	116 (49.7)
RDW	
Mean (SD)	12.5 (1.0)
MPV	
Mean (SD)	8.0 (1.2)
Fibrinogen	
Mean (SD)	3.3 (0.9)

Univariate analysis showed that NLR, MLR, pathological tumor size, perineural/perivascular invasion and differentiation grade were significant risk factors for cervical lymph metastasis in cN0 OSCC of the tongue (Table 2). 

**Table 2:** Univariate analysis of risk factors for occult neck metastasis. SD: standard deviation; t: Student t; U: Mann-Whitney U; χ2: Chi-squared test; df: degrees of freedom; NLR: neutrophil-lymphocyte ratio; MLR: monocyte-lymphocyte ratio; PLR: platelet-lymphocyte ratio.

Univariate analysis	Occult neck metastasis		Test value	df	p-value
	Yes (n=26)	No (n=76)			
Age - mean (range)	62.8 (42-87)	59.2 (24-89)	1.1t	100.0	0.268
NLR - mean (SD)	3.0 (1.7)	2.0 (0.9)	570U		0.001
MLR - mean (SD)	0.3 (0.2)	0.2 (0.1)	657U		0.011
PLR - mean (SD)	129.2 (60.1)	111.4 (45.1)	780U		0.111
RDW - mean (SD)	12.4 (1.0)	12.5 (1.0)	923U		0.620
MPV - mean (SD)	8.2 (1.3)	7.9 (1.1)	829U		0.221
Fibrinogen - mean (SD)	3.5 (0.1)	3.2 (0.9)	120U		0.608
Gender					
Male - n (%)	16 (15.7)	47 (46.1)	7.56e-4 χ2	1	0.978
Female - n(%)	10 (9.8)	29 (28.4)			
Tumor dimension					
p<2 cm - n (%)	5 (4.9)	33 (32.4)	4.85 χ2	1	0.028
p 2-4 cm - n (%)	21 (20.6)	43 (42.2)			
Perineural/perivascular invasion					
Yes - n (%)	14 (13.7)	11 (10.8)	16.2 χ2	1	<0.01
No - n (%)	12 (11.8)	65 (63.7)			
Differentiation grade					
G1 - n (%)	1 (1.0)	19 (18.6)	7.0 χ2	2	0.030
G2 - n (%)	20 (19.6)	51 (50.0)			
G3 - n (%)	5 (4.9)	6 (5.9)			

Multivariate analysis was performed on NLR, MLR, pathological tumor size, perineural/perivascular invasion and differentiation grade. Results showed that only NLR and perineural/perivascular invasion were independent risk factors for cervical lymph node metastasis (Table 3).

**Table 3:** Multivariate analysis of risk factors for occult neck metastasis. OR: odds ratio; CI: confidence interval; NLR: neutrophil-lymphocyte ratio.

Multivariate analysis	Test value	SE	OR (95% CI)	p-value
NLR	3.1	0.2	1.9 (1.2-2.9)	0.002
Perineural/perivascular invasion	3.6	0.5	7.2 (2.5-20.8)	<0.001
R2= 0.2				

Given the univariate and multivariate analysis results, we examined the relationship between NLR and occult neck metastasis with further depth. To assess and quantify the discriminatory performance of NLR in predicting neck status classification, we performed a ROC curve analysis, that revealed an area under the curve of 0.712. Younden’s Index determined the NLR value of 2.96 as the most adequate cut-off value for neck status prediction.

As a secondary aim of the study, given the previously reported univariate and multivariate analysis results, and the importance of DOI in all the recent therapeutic decision guidelines, we decided to assess the relationship between NLR and primary tumor DOI. Unfortunately, following a tendency that is not uncommon globally, in Instituto Português de Oncologia de Lisboa, DOI has only been systematically recorded in all histopathological specimens after 2018, following the publication of the 8th edition of the American Joint Committee on Cancer staging manual [18]. Therefore, in our cohort of patients, only 33 individuals had a record of DOI on postoperative histopathological examination. Nevertheless, we divided that group of patients into two subgroups - DOI < 4 mm and DOI ≥ 4 mm - and analyzed the relationship with NLR values. Statistical analysis showed that NLR values of pretreatment workup had a significant association with the DOI on postoperative histopathological examination (Table 4).

**Table 4:** Relation between preoperative workup NLR value and DOI of the primary tumor. U: Mann-Whitney U; NLR: neutrophil-lymphocyte ratio; DOI: depth of invasion.

NLR and DOI association	Depth of invasion			
	<4 mm (n=6)	>4 mm (n=27)	Test value	p-value
NLR (mean, SD)	1.4 (0.3)	2.8 (1.8)	31U	0.018

## Discussion

The present study supports the impact of pretreatment NLR on the prediction of occult neck metastasis and suggests a potential application of this ratio on the prediction of primary tumor DOI. However, we did not find any significant associations between the other studied pretreatment inflammatory blood markers and occult neck metastasis.

The approach to patients with early-stage OSCC and with cN0 has been a matter of controversy for several years [2,5]. Although there is a lack of global consensus amongst the published guidelines on the management of cN0 neck, there has been a growing trend in the literature towards upfront END in opposition to a “watch and wait policy” [2,7]. That tendency can be confirmed by recent large-scale studies that demonstrate a clear benefit in disease-free survival and overall survival with upfront END [19,20]. Despite all the evidence, it is clear that patients without occult neck disease, who undergo END are afforded no therapeutic benefit and may incur additional morbidity from treatment [5].

Sentinel Node Biopsy has been described as an alternative to END, for identifying occult neck metastasis in patients with early-stage OSCC [7,21,22] Nevertheless, procedural success rates for sentinel node identification and the accuracy in detecting occult lymphatic metastasis depend on technical expertise and experience [7]. On the other hand, despite evidence that Sentinel Node Biopsy benefits true pN0 patients, by sparing them the potential morbidity of neck dissection, its application in pN+ patients might prove deleterious, as it is not therapeutic for this group and exposes patients to two procedures, potentially leading to a delay in delivery of adjuvant treatment [2].

The imaging methods most commonly used for preoperative assessment of lymph node status are CT, MRI (magnetic resonance imaging), US and positron emission tomography [2,5]. Due to superior anatomic resolution, CT and MRI remain the standard for assessing cervical lymph node status at presentation [5]. Nevertheless, large studies have demonstrated the limitation of any preoperative imaging in accurately staging the clinically negative neck [2,23,24].

Various references have demonstrated the role of histopathologic parameters of the primary tumor, such as tumor size [25-27], DOI [25,26,28], tumor thickness [25,27,28], differentiation grade [25,26], perivascular/perineural invasion [27,29] as possible predictors of occult neck metastasis. Our study confirms the role of tumor size, differentiation grade and perivascular/perineural invasion in accurately staging the clinically negative neck in early-stage OSCC of the tongue.

In recent years DOI has gradually been gaining importance over other primary tumor histopathologic parameters, with numerous reports showing a strong positive correlation between DOI and nodal metastasis [30-32]. The high prognostic significance of DOI in staging OSCC, in predicting nodal metastasis and dictating the management of the neck has been reflected in its inclusion in numerous position-papers and guidelines [2,7] as well as in the latest 8th AJCC classification update [18]. Recent evidence suggests that the DOI threshold beyond which the risk of nodal metastasis increases is 1.5 mm for floor of the mouth tumors and 4 mm for the remaining oral cavity [2,33,34].

Unfortunately, despite all data concerning the role of histopathologic parameters of the primary tumor, in most clinical scenarios, that data is not available to the surgeon, before the surgical procedure, which jeopardizes its practical application. As an example, it is well established that mainly in superficial tumors the clinical assessment of DOI, by tumor palpation has several limitations [35], and that even MRI imaging only moderately correlates with tumor DOI [35] and tumor thickness [36]. Therefore, any additional tools that could be developed to complement the described clinical, histopathological and imaging resources would help clarify the management of OSCC cN0.

In the past three decades, the relation between cancer and the immune system has been increasingly recognized, with chronic inflammation being described as an established risk factor for developing several types of tumors [37,38]. It was also shown that circulating hematopoietic stem and progenitor cells in cancer are predominantly myeloid-based over hematopoietic precursors with lymphoid potential, with a preferential expansion of granulocytic precursor cells [37,39]. On those grounds, several reports have indeed shown a negative prognostic value of higher NLR ratios in multiple sites of head and neck [37,40-42] cancer. Two studies have also assessed the usefulness of NLR in predicting which patients present a higher risk of occult neck metastasis [3,6].

Other inflammatory biomarkers have also been described as prognostic factors for several malignancies such as MLR [10,11], PLR [15,42], RDW [12], MPV [43] and fibrinogen [12]. Therefore, in our study, we decided to assess the predictive ability of such inflammatory biomarkers in accurately staging the clinically negative neck, additionally to NLR. To the best of our knowledge, this is the first report evaluating the capability of these inflammatory biomarkers, in predicting occult neck metastasis in early-stage OSCC of the tongue. On univariate analysis, we found a significant association between NLR and MLR, with occult neck metastasis. Nevertheless, on multivariate analysis, NLR was the only inflammatory biomarker that was an independent risk factor for neck status. This finding could be explained by the already described skewing of the cell population balance towards an increase of myeloid and a decrease of lymphoid cell populations [37]. Other than that, our findings seem to unveil a certain predominance of neutrophil activity on cancer progression towards regional metastasis. That is consistent with other studies reporting that neutrophils may facilitate tumor growth and the formation of metastasis through a various number of mechanisms like: the release of soluble factors and proteases, such as prostaglandin E2 and neutrophil elastase [37,44]; production of pro-angiogenic factors, such as metalloproteinase-9 and vascular endothelial growth factor [9]; or the inhibition of effector T-cells and natural killer cells and modulation of macrophage activity [9,45].

Our study confirmed a clear association between NLR values and neck status in early OSCC of the tongue. We obtained a cut-off value of 2.96, which interestingly is very close to the cut-off values of the studies that also tried to clarify the relation between NLR and occult neck metastasis, by Wu, et al. (2.95) and Abbate, et al. (2.93), notwithstanding the differences in statistical analysis.

Only 33 of our patients had DOI values on their histopathological reports because that parameter has only been recorded systematically in the center where we conducted this research, after the publication of the 8th edition of the American Joint Committee on Cancer staging manual [18]. Nevertheless, we were quite curious about the relation between pretreatment NLR and primary tumor DOI, and defined that as a secondary aim of this study. Therefore we divided the group of patients that had a DOI record into two subgroups based on the well-established 4 mm DOI cut-off value [33,34], and we obtained a significant association between higher NLR values and the “deeper” tumors. Other studies have found a similar association between tumor thickness and NLR [3]. Still, to the best of our knowledge, this study is the first to assess the association between NLR and DOI in head and neck cancer.

Some limitations of our research should be mentioned. First of all, we have to consider that our work’s statistical power was decreased by the inherent bias of a retrospective study in a single center.

We also decided not to analyze the association of our patients smoking and alcohol consumption patterns due to the heterogeneity of data recording and missing information that we found in patient’s records regarding those habits. On the other hand, human papillomavirus status is not usually assessed on a routine basis for OSCC. Obviously, the absence of those variables (smoking and drinking habits, and human papilloma virus status) in our analysis can lead to a bias, since they are well-known risk factors for OSCC. Nevertheless, the other two studies [3,6] already conducted to clarify the role of NLR with occult neck metastasis did not have any record of human papillomavirus status, and did not perform any statistical analysis of the relation between smoking and drinking habits and the development of occult neck metastasis. However, the study by Wu et al. [3], examined the association between NLR values with smoking and drinking habits, but no significant association was found. 

Although all patients with chronic inflammatory disease have been discarded through our exclusion criteria, we have to carefully interpret our results due to the well-established interference that the immune and inflammatory condition of the patient, at the time of blood withdrawal, may have played on our study results.

However, NLR has several advantages: it is easily and objectively measured, cost-effective, and always available before treatment planning [46]. This study demonstrates quite obviously the role of NLR in predicting occult neck metastasis with a well-defined cut-off value and with clearly determined sensitivity and specificity values. In our model, the 2.96 NLR value was not a sensitive tool to predict the cN0 (sensitivity 53.9%) neck status, but by contrast, was a specific predictor (specificity 89.5%) for that matter. We can obtain a reference point towards the potential role of NLR if we look at evidence that the specificity of the imaging techniques most commonly used in assessing occult neck metastasis (US, MRI, CT and positron emission tomography) range from 62.5% to 79.4% [47].

In practical terms, NLR could be a useful complementary tool for clinical decision making, in centers where sentinel node biopsy is not available in patients with cN0 early-stage OSCC of the tongue, when neck imaging is equivocal in detecting metastatic lymph nodes. In those situations, if a value of NLR <2.96 was recorded in the pretreatment workup, there would be an 85% (negative predictive value) probability of a pN0 neck.

This study also indicates that NLR could also play a role in helping the surgeon in differentiating between “thin” (DOI<4 mm) and “thick” tumors (DOI≥4 mm), which is a significant factor in clinical decision of OSCC neck management according to most of the recent guidelines [2,7]. However, only 33 patients of our sample had a DOI histopathological record.

## Conclusions

Our study supports the role of pretreatment NLR in predicting occult neck metastasis in early stage OSCC of the tongue. It also raises the question about the potential of NLR as a predictor of the primary tumor’s DOI. Further studies on larger series are needed to determine the cut-off value of NLR as a predictor of occult neck metastasis in other populations and to clarify the association of NLR with primary tumor’s DOI.
